# Vaspin Alleviates Sepsis-Induced Cardiac Injury and Cardiac Inflammation by Inhibiting Kallikrein 7 in Mice

**DOI:** 10.1155/2022/1149582

**Published:** 2022-07-15

**Authors:** Na Yin, Fuze Pan, Lingyue Qiu, Zicong Yang, Rixin Xiong, Lei Shi, Ying Shi, Ning Wu, Kui Wu, Qingkuan Li, Daojun Wen, Qili Huang, Yuyan Zhang, Yuhong Mi, Qingwei Ji

**Affiliations:** ^1^Emergency Critical Care Center, Beijing Anzhen Hospital, Capital Medical University, Beijing, China; ^2^Department of Cardiology, The People's Hospital of Guangxi Zhuang Autonomous Region, Nanning, China; ^3^Institute of Cardiovascular Sciences, Guangxi Academy of Medical Sciences, Nanning, China

## Abstract

**Background:**

Vaspin is an important adipokine that is involved in cardiovascular diseases. This study is aimed at investigating whether vaspin participates in sepsis-induced cardiac injury and explored the possible mechanism.

**Methods:**

First, cecal ligation and puncture (CLP) and lipopolysaccharide (LPS) were used to establish a mouse model of sepsis, and cardiac vaspin expression was examined. In addition, after pretreatment with vaspin or phosphate-buffered saline (PBS), wild-type (WT) mice underwent CLP to establish a septic model and received sham as a control. Finally, WT mice and kallikrein 7 (KLK7-/-) mice were underwent CLP with or without vaspin pretreatment.

**Results:**

Mice that underwent CLP and were administered LPS exhibited increased vaspin expression in both the heart and serum compared with sham- or saline-treated mice. In CLP mice, pretreatment with vaspin reduced mortality and alleviated the expression of cardiac injury markers and cardiac dysfunction. In addition, vaspin reduced the cardiac levels of CD45+ cells and CD68+ cells, alleviated the cardiac inflammatory response, and reduced cardiomyocyte apoptosis. The protective effects of vaspin on CLP mice were masked by the deletion of KLK7, which was demonstrated to be a downstream signal of vaspin.

**Conclusions:**

Vaspin alleviates cardiac inflammation and plays a protective role in sepsis-induced cardiac injury by reducing KLK7 expression.

## 1. Introduction

Sepsis is a systemic inflammatory syndrome caused by the invasion of pathogenic microorganisms in the body that affects approximately 18 million people every year and leads to 14,000 deaths every day [1]. The process of sepsis is often accompanied by the release of lipopolysaccharide (LPS), which leads to the activation of a variety of immune cells and a variety of pathological reactions [2, 3]. Then, it causes tissue and organ injury and failure, which is an important cause of death in patients, among which heart failure is the most important cause of death due to sepsis [2, 3]. Therefore, it is critical to identify potential intervention targets to prevent sepsis-induced cardiac injury and cardiac dysfunction.

Vaspin, also known as SerpinA12, is a member of the Serpin family of serine protease inhibitors [4]. Vaspin is an adipokine that is mainly derived from visceral fat cells and can be expressed in a variety of tissues, such as the heart, kidney, brain, gastrointestinal tract, and pancreas [5]. Inflammation, high glucose, and other pathological conditions can promote the synthesis and secretion of vaspin, and vaspin can alleviate the inflammatory response in tissues and organs by inhibiting kallikrein 7 (KLK7), which was the first discovered downstream signaling pathway [6].

Numerous studies have shown an association between vaspin and the progression of cardiovascular diseases. Treatment with vaspin can significantly protect against methylglyoxaldehyde-induced apoptosis in human umbilical vein endothelial cells and delay high glucose-induced vascular smooth muscle cell proliferation and migration [7, 8]. Vaspin is increased in coronary artery disease (CAD) patients and positively correlates with the severity of CAD [9, 10]. Vaspin is also increased in atherosclerotic mice and reverses atherosclerosis progression in ApoE-/- mice by alleviating inflammation [10–12]. However, it remains unclear whether vaspin plays a role in sepsis-induced cardiac injury, and we examined this question using mice with cecal ligation and puncture- (CLP-) induced sepsis in this study.

## 2. Experimental Materials and Methods

### 2.1. Mice and Sepsis Model

**Figure 1:**
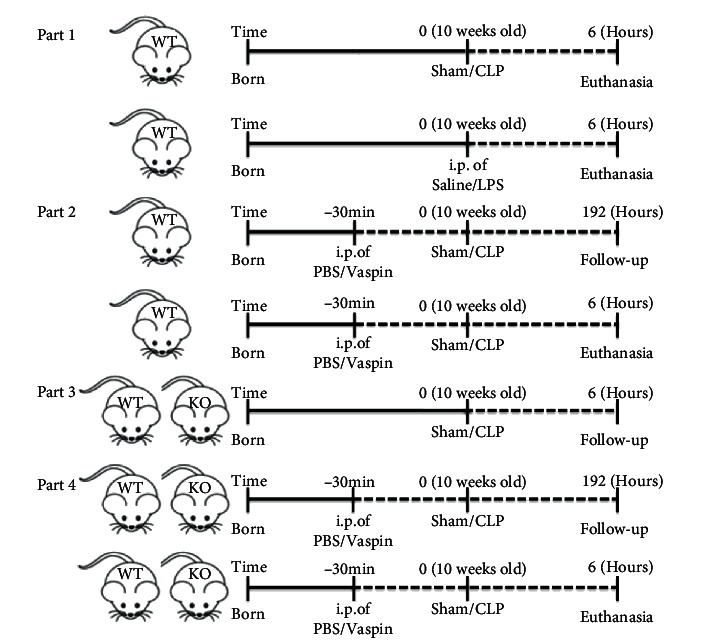
Establishment and pretreatment of the mouse sepsis model.

Wild-type (WT) mice and kallikrein 7 knockout (KLK7-/-) mice on a C57BL/6 background were purchased from GemPharmatech Co., Ltd. (Jiangsu, China). Male WT mice and KLK7-/- mice aged 10 weeks were used in this study. First, WT mice were subjected to CLP or stimulated with LPS (10 mg/kg, Sigma) to establish a mouse model of sepsis as described in previous studies [13, 14]. Six hours later, vaspin expression was analyzed, while WT mice underwent a sham operation or were administered saline as a control (*n* = 5 for each group, part 1). Additionally, before being subjected to CLP or a sham operation, WT mice were pretreated with PBS or vaspin (0.1 *μ*g/kg, PeproTech) for 30 minutes as previously described [15], and then, the effects of vaspin on cardiac injury were detected (*n* = 10 − 12 in each group); some mice were observed for 8 days to determine the survival rates in each group (part 2). Third, WT mice and KLK7-/- mice underwent CLP or sham for the analysis of cardiac inflammation (*n* = 5 in each group, part 3). Finally, WT mice and KLK7-/- mice were pretreated with vaspin or PBS and then underwent CLP and observed for 6 hours or 8 days to determine whether vaspin affects sepsis-induced cardiac injury and survival rates by regulating KLK7 (*n* = 10 − 13 in each group, part 4). The treatment of mice and the establishment of the sepsis model are shown in Figure 1. This study was approved by our institution's Ethics Committee (approval no. Cardiac2020-01-122).

### 2.2. Implementation of CLP

CLP was performed as described in previous studies [16, 17]. Briefly, WT mice or KLK7-/- mice were pretreated with vaspin or PBS, anesthetized with 2% isoflurane inhalation, and laid flat on a preheated operating table. The abdominal hair was removed, 75% ethanol was used to disinfect the skin, and the abdominal skin was dissected to expose the abdominal cavity. The cecum was ligated at half the distance between the distal pole and the base of the cecum and then punctured once from the mesenteric end in the antimesenteric direction using a 26 G needle. Finally, the abdominal skin was sutured and disinfected, and the mice were placed in an incubator at 28°C for resuscitation.

### 2.3. Examination of Cardiac Function

After 6 hours of CLP or sham operation, the mice were anesthetized as described above and placed flat on the operating table. A MyLab™30CV ultrasound system (Esaote SpA, Genoa, Italy) consisting of a 10 MHz linear array ultrasonic transducer was used to collect data related to left ventricular (LV) structure and function, including LV end-systolic diameter (LVESD), LV end-diastolic diameter (LVEDD), ejection fraction (EF), and fractional shortening (FS). A Millar Pressure Volume system containing a microtip catheter transducer (Millar, Inc.) was used to collect the LV signals. The microtip catheter transducer was inserted into the left ventricle via the right carotid artery, and information on the maximal slope of the systolic pressure increment (+dp/dt max) and diastolic pressure decrement (-dp/dt max) were recorded on a beat-by-beat basis.

### 2.4. Analysis of Cardiac Injury Markers

Blood samples were collected and centrifuged to obtain serum. The levels of cardiac injury markers, including lactate dehydrogenase (LDH) and myocardial-bound creatine kinase (CK-MB), were examined using LDH assay kits and CK-MB assay kits (both purchased from Njjcbio Biotechnology, China), respectively, according to the manufacturer's instructions.

### 2.5. Western Blot Analysis

The frozen LV tissue was ground, lysed with lysis buffer, and further lysed by ultrasound. Total protein was obtained by centrifugation, the supernatants were collected, and all samples were quantified using a BCA protein quantification kit (Thermo Fisher Scientific, USA) and adjusted to the same concentration. Total protein was separated by molecular weight by electrophoresis using 10% Laemmli sodium dodecyl sulfate (SDS) polyacrylamide gels and transferred to Immobilon-FL PVDF membranes (Millipore, USA). After blocking with 3% BSA for 1 hour, the membranes were incubated with rabbit antimouse vaspin (1 : 1000), rabbit antimouse KLK7 (1 : 1000), rabbit antimouse nuclear factor-kappa B p65 (1 : 1000), rabbit antimouse nuclear factor-kappa B (NF-*κ*B) p-p65 (1 : 500), rabbit antimouse Bax (1 : 1000), rabbit antimouse Bcl2 (1 : 500), and rabbit antimouse GAPDH (1 : 2000, all monoclonal antibodies were purchased from Abcam, UK) overnight (9 hours) to examine target protein expression. After incubation at room temperature for 1 hour with the secondary antibody, the membranes were scanned using an Odyssey system.

### 2.6. Real-Time Quantitative Polymerase Chain Reaction (RT–qPCR)

LV tissue was lysed in TRIzol reagent (Roche, Germany), total RNA was extracted, and the concentration of each sample was determined. Two micrograms of total RNA from each sample was reverse transcribed into cDNA using a reverse transcription kit (Roche, Germany) according to the manufacturer's instructions. Then, the forward primer, reverse primer, and LightCycler 480 SYBR Green Master Mix (Roche, Germany) were used for PCR amplification to measure the expression of target genes, including interleukin-6 (IL-6), IL-17, tumor necrosis factor *α* (TNF-*α*), interferon *γ* (IFN-*γ*), and monocyte chemoattractant protein-1 (MCP-1). Target gene expression was normalized to GAPDH. The RT-qPCR primer sequences were as described in our previous study [18].

### 2.7. Histological Analysis

Hearts were fixed in 4% neutral paraformaldehyde for 4 days. After paraffin embedding, the hearts were cut to a thickness of 4 *μ*m and then transferred and fixed on slides. The slides were incubated with anti-CD45 and anti-CD68 antibodies (both purchased from GeneTex, USA) to examine the levels of cardiac leukocytes and macrophages. Apoptotic cells in the left ventricle were examined by terminal deoxynucleotidyl transferase-mediated dUTP nick end labeling (TUNEL) staining (Millipore, USA) using a commercially available kit according to the manufacturer's instructions. In addition, cardiomyocytes were incubated with anticleaved caspase3 (Abcam, UK) to examine the effects of vaspin on cardiomyocyte apoptosis.

### 2.8. Cell Studies and Analysis

Cardiomyocytes were isolated as previously described [19]. Briefly, WT mice and KLK7-/- mice aged 8 weeks were euthanized, the chest cavity was exposed, and the hearts were quickly collected. The hearts were rinsed with phosphate-buffered saline (PBS), and the left ventricle was further isolated, gently prepared into 1 mm^3^ fragments, and then digested with mixed enzymes, including EDTA buffer, perfusion buffer, and collagenase buffer (7 : 3 : 40, triple purchase form Servicebio, China). After being passed through a 100 *μ*m filter, the cells underwent 4 sequential rounds of gravity settling, and then, 3 intermediate calcium reintroduction buffers were used to gradually restore the calcium concentration to physiological levels. Then, WT cardiomyocytes and KLK7-/- cardiomyocytes were obtained.

RPMI 1640 medium (Gibco, USA) with 10% fetal bovine serum was used to culture the cardiomyocytes. First, WT cardiomyocytes were pretreated with PBS or vaspin (80 ng/ml, PeproTech, USA) and then administered saline or LPS (1 *μ*mol/ml, Sigma, USA) [13, 20]. Additionally, WT and KLK7-/- cardiomyocytes were pretreated with vaspin or PBS and then stimulated with LPS. All cardiomyocytes were cultured for 24 hours, the culture medium was changed once every 8 hours, and both vaspin and LPS were added again. Cleaved caspase-3 expression was examined as described above.

### 2.9. Statistical Analysis

All data in this study were analyzed using GraphPad Prism 8 software and are presented as the mean ± SD. Differences between 2 groups or multiple groups were compared using Student's *t* test or one- two-way ANOVA, respectively. The log-rank test was used to analyze the differences in the survival rates among the four groups at follow-up. A value of *p* < 0.05 was considered statistically significant.

## 3. Results

### 3.1. Vaspin Expression Is Increased in Septic Mice

**Figure 2:**
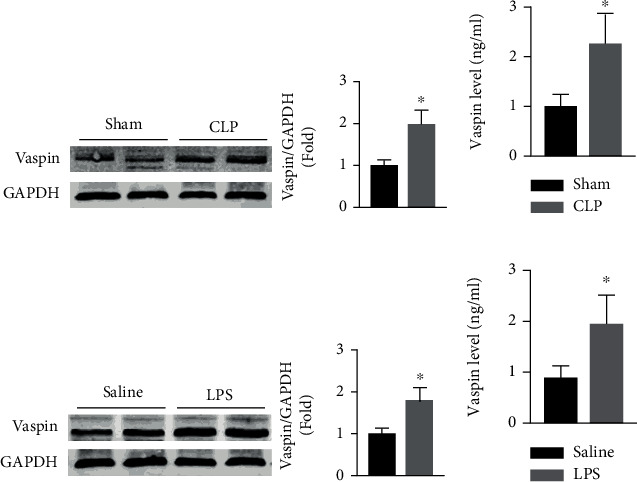
Effects of sepsis on vaspin expression in mice. Cardiac (a) and serum (b). Vaspin expression in sham and CLP mice (Student's *t* test). Cardiac (c) and serum (d). Vaspin levels in saline- and LPS-treated mice (Student's *t* test). *N* = 6 in each group. ^∗^*p* < 0.05 vs. the sham or saline group.

In the CLP-induced mouse model of sepsis, cardiac and serum vaspin levels were, respectively, increased 0.98-fold and 1.33-fold compared with those in sham mice (Figures 2(a) and 2(b)). And the magnitude of increase was 0.79-fold and 1.19-fold in the LPS-induced mouse sepsis model (Figures 2(c) and 2(d)).

### 3.2. Treatment with Vaspin Alleviates Cardiac Injury and Cardiac Dysfunction in CLP-Induced Septic Mice

**Figure 3:**
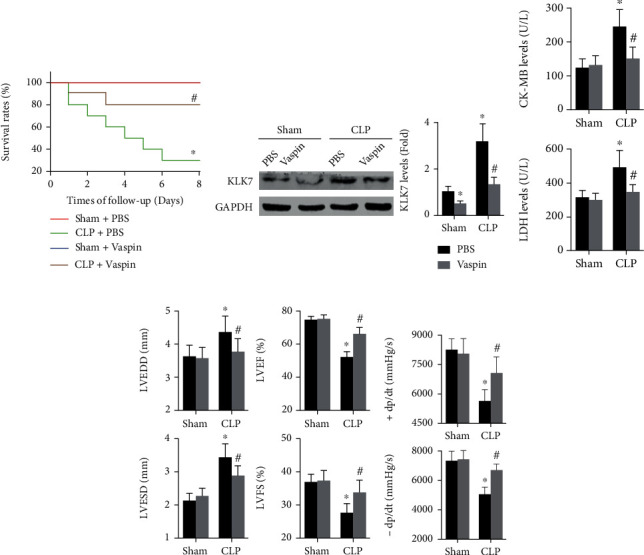
Effects of vaspin on survival rates and cardiac dysfunction in CLP mice. (a) The survival rates in each group were examined during the 8-day follow-up (log-rank test). (b) Cardiac KLK7 expression in the four groups was measured (two-way ANOVA). (c) Serum levels of CK-MB and LDH in all groups were analyzed (two-way ANOVA). (d, e) Cardiac structural and functional data were examined, including LVEDD, LVESD, LVEF, FS, +dp/dt max, and -dp/dt max (two-way ANOVA). *N* = 5 − 10 in each group. ^∗^*p* < 0.05 vs. the sham + PBS group. ^#^*p* < 0.05 vs. the CLP + PBS group.

During the 8-day follow-up, pretreatment with vaspin did not affect the survival rates of sham mice but improved the survival rates of septic mice (CLP + PBS group: 30.0% vs. CLP + vaspin group: 80.8%, Figure 3(a)). Vaspin decreased cardiac KLK7 expression for 49% in sham and for 57% in CLP mice (Figure 3(b)). In addition, vaspin decreased LDH levels for 38% and reduced CK-MB levels for 29.7% in septic mice that underwent CLP for 5 days (Figure 3(c)). Furthermore, treatment with vaspin alleviated sepsis-induced cardiac dysfunction, as indicated by reductions in LVEDD, LVESD, +dp/dt max, and -dp/dt max, as well as increases in LVEF and FS (Figures 2(d) and 2(e)).

### 3.3. Vaspin Alleviates Sepsis-Induced Cardiac Inflammation in Mice

**Figure 4:**
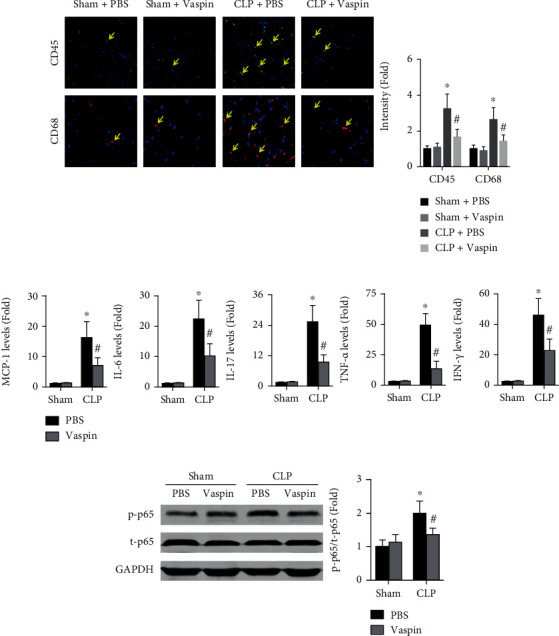
Effects of vaspin on sepsis-induced cardiac inflammation in mice. (a) Cardiac CD45+ cells and CD68+ cells in each group were examined (two-way ANOVA). (b) Cardiac mRNA expression levels of MCP-1, IL-6, IL-17, TNF-*α*, and IFN-*γ* were measured (two-way ANOVA). (c) The phosphorylation of NF-*κ*B p65 in each group (two-way ANOVA). Blue, green, and red indicate nuclei, CD45-positive cells, and CD68-positive cells, respectively. *N* = 5 in each group. ^∗^*p* < 0.05 vs. the sham + PBS group. ^#^*p* < 0.05 vs. the CLP + PBS group.

The effects of vaspin on inflammation were investigated, and the results showed that pretreatment with vaspin decreased the cardiac infiltration of CD45+ cells and CD68+ cells (Figure 4(a)). The expression levels of various inflammatory markers, including MCP-1, IL-6, IL-17, TNF-*α*, and IFN-*γ*, were decreased by approximately 45-70% after vaspin treatment (Figure 4(b)). In addition, vaspin decreased the phosphorylation of NF-*κ*B p65 in septic mice (Figure 4(c)).

### 3.4. Vaspin Protects against Cardiomyocyte Apoptosis in Septic Mice

**Figure 5:**
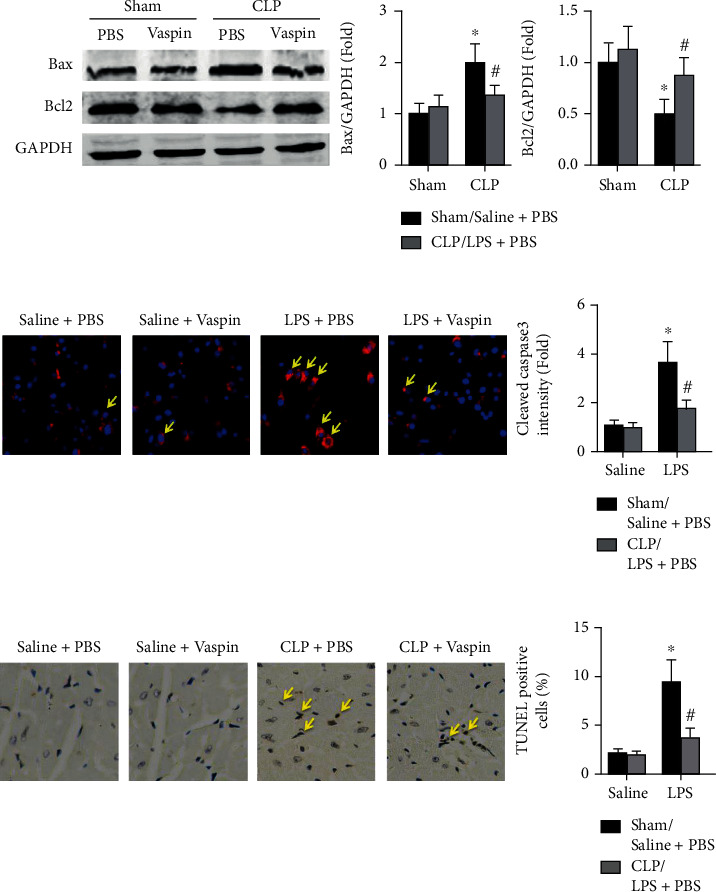
Effects of vaspin on cardiomyocyte apoptosis in septic mice. (a) Protein expression levels of cardiac Bax and Bcl2 were examined (two-way ANOVA). (b) Cleaved caspase-3 expression in LPS-induced cardiomyocytes was analyzed (two-way ANOVA). Blue and red spots indicate the nucleus and cleaved caspase3. (c) TUNEL-positive cells in each group were determined (two-way ANOVA). *N* = 5 in each group. ^∗^*p* < 0.05 vs. the sham + PBS or saline + PBS group. ^#^*p* < 0.05 vs. the CLP + PBS or LPS + PBS group.

Cardiomyocyte apoptosis was then examined, and the results showed that treatment with vaspin increased cardiac expression levels of Bcl2 for 88% in septic mice but decreased Bax expression for 34% (Figure 5(a)). Reduced levels of cleaved caspase3 were observed in LPS-stimulated WT cardiomyocytes when vaspin was added in vitro (Figure 5(b)). Pretreatment also decreased the number of TUNEL-positive cardiac cells in septic mice (CLP + PBS group: 9.42 ± 2.43 vs. CLP + PBS group: 3.67 ± 0.64, Figure 5(c)).

### 3.5. KLK7 Knockout Mediates the Effects of Vaspin on Sepsis-Induced Cardiac Injury and Cardiac Dysfunction

**Figure 6:**
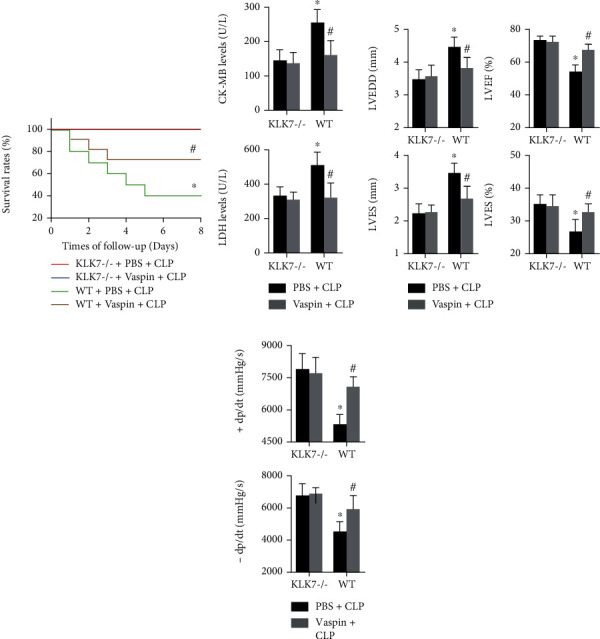
Effects of KLK7 deficiency on sepsis-induced cardiac injury in mice. (a) The survival rates of septic WT mice and KLK7-/- mice were measured (log-rank test). (b) CK-MB and LDH concentrations in serum were examined (two-way ANOVA). (c, d) Cardiac structure and function were measured (two-way ANOVA). *N* = 5 in each group. ^∗^*p* < 0.05 vs. the WT + PBS + CLP group. ^ns^*p* > 0.05 vs. the KLK7 − /−+PBS + CLP group.

The effects of vaspin on the survival rates of KLK7-/- mice were examined, and the results showed that none mice died in septic KLK7-/- mice with or without vaspin treatment, and vaspin had no effects on the survival rates of KLK7-/- mice (Figure 6(a)). Vaspin did not affect cardiac injury marker expression in KLK7-/- mice (Figure 6(b)). The regulatory effects of vaspin on cardiac function were also evident in septic KLK7-/- mice (Figures 6(c) and 6(d)).

### 3.6. KLK7 Deficiency Masks the Anti-Inflammatory and Protective Effects of Vaspin on Septic Mice

**Figure 7:**
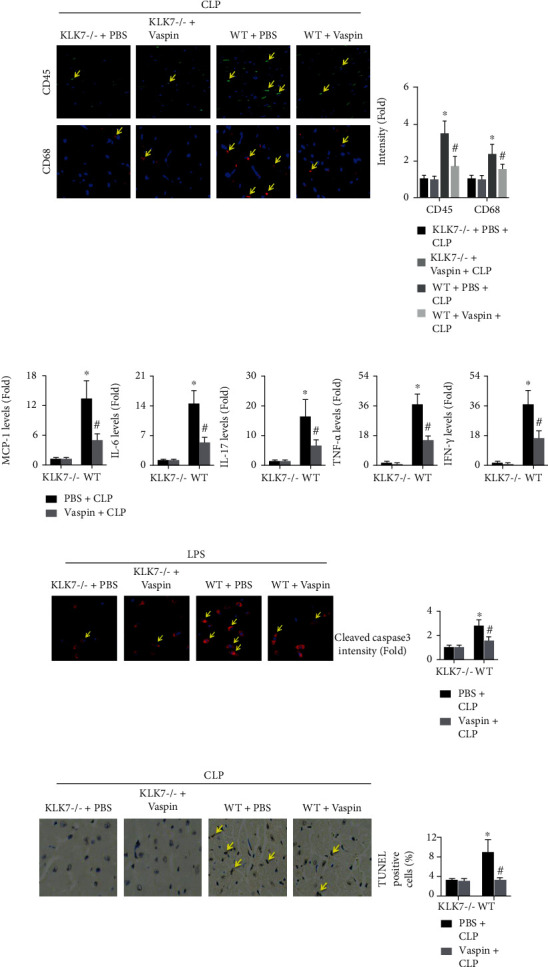
Effects of KLK7 deletion on cardiac inflammation and cardiomyocyte apoptosis in septic mice. (a, b) Levels of CD45+ cells and CD68+ cells, as well as the levels of the proinflammatory cytokines MCP-1, IL-6, IL-17, TNF-*α*, and IFN-*γ* in the heart (two-way ANOVA). Blue, green, and red indicate nuclei, CD45-positive cells, and CD68-positive cells, respectively. (c) The expression of cleaved caspase3 in LPS-treated WT cardiomyocytes and KLK7-/- cardiomyocytes was analyzed (two-way ANOVA). Blue and red spots indicate the nucleus and cleaved caspase3, respectively. (d) The number of TUNEL-positive cardiac cells was quantified (two-way ANOVA). ^∗^*p* < 0.05 vs. the WT + PBS + CLP or WT + PBS + LPS group. ^ns^*p* > 0.05 vs. the KLK7 − /−+PBS + CLP or KLK7 − /−+PBS + LPS group.

Cardiac inflammation was examined, and the results showed that KLK7 knockout significantly improves cardiac inflammation response in septic mice, but still higher than the sham+KLK7-/- group (as the supplemental Figure S1). While treatment with vaspin did not decrease the number of CD45+ cells or CD68+ cells in the hearts of septic KLK7-/- mice (Figure 7(a)). Pretreatment with vaspin did not affect the cardiac expression of MCP-1, IL-6, IL-17, TNF-*α*, or IFN-*γ* in CLP mice (Figure 7(b)). No changes in cleaved caspase3- or TUNEL-positive cell percentages were observed in vaspin-treated KLK7-/- mice (TUNEL levels, CLP + KLK7 − /−+PBS group: 3.11 ± 0.45 vs. CLP + KLK7 − /−+vaspin group: 2.98 ± 0.31, Figures 7(c) and 7(d)).

## 4. Discussion

In this study, we examined the regulatory role of the adipokine vaspin in cardiac injury induced by sepsis and focused on the inflammatory response in the heart to elucidate the mechanism. We showed for the first time that vaspin alleviates cardiac inflammation, reduces cardiomyocyte apoptosis, improves cardiac injury, and increases the survival rates of septic mice. These effects were masked when KLK7 was knocked out. Our findings suggest that vaspin can reduce sepsis-induced cardiac inflammation, alleviate sepsis-induced cardiac injury, and improve cardiac function by inhibiting KLK7 expression. Vaspin may be a therapeutic target for the prevention and treatment of cardiac injury in clinical sepsis.

Sepsis is a major public health problem worldwide and is a systemic inflammatory response syndrome caused by the invasion of bacteria and other pathogenic microorganisms in the body [21, 22]. The cell wall of gram-negative bacteria contains LPS, which can be released into the blood after infection and lead to inflammatory reactions, oxidative stress, coagulation dysfunction, immune system activation, and other pathological processes and then induce tissue and organ injury, which suggests that LPS is the initiating factor in the occurrence and development of sepsis [22, 23]. In addition, a previous study reported that circulating vaspin levels were increased in septic patients [24]. Thus, in this study, mice underwent CLP to induce abdominal infection and simulate clinical sepsis, while intraperitoneal injection of LPS was used to simulate the release of LPS from the cell walls of gram-negative bacteria. Therefore, mice underwent CLP and were administered LPS to establish sepsis models, and vaspin expression was examined to determine the effect of CLP on vaspin expression. The results showed that the expression of cardiac vaspin was significantly increased in CLP- and LPS-treated mice. These findings suggest that vaspin may be involved in sepsis-induced cardiac injury, while the effects of vaspin on cardiac injury need to be further determined. In subsequent experiments, we examined the effects of vaspin on mortality and cardiac injury in CLP-induced septic mice, and the results showed that pretreatment with vaspin significantly increased the survival rate, decreased the expression of multiple markers of cardiac injury, and improved cardiac dysfunction. These results suggest that vaspin was compensatorily increased to protect against sepsis-induced cardiac injury. Just as B-type natriuretic peptide (BNP), which can play a vein dilating and diuretic role, is compensatory increased in heart failure and beneficial in improving symptoms, BNP-related drugs are used in the treatment of clinical chronic heart failure [25].

In sepsis patients and mice, it was found that the levels of various immune cells in the blood, including leukocytes, monocytes, and macrophages, were significantly increased. In addition, these immune cells gradually infiltrate the heart and become activated, thus releasing multiple factors and amplifying the cardiac inflammatory response [26–28]. These findings suggest that inflammation plays an important role in the progression of sepsis, although other pathological factors, including oxidative stress and vascular injury, are involved in sepsis progression. Therefore, to investigate the mechanism by which vaspin protects against sepsis-induced cardiac injury, cardiac inflammation in each group of mice was investigated. The results showed that vaspin significantly reduced the levels of cardiac leukocytes and macrophages and downregulated the expression of several proinflammatory factors. These results suggest that vaspin alleviates sepsis-induced cardiac dysfunction by reducing cardiac inflammation.

The antiapoptotic protein Bcl2 is present in the outer mitochondrial membrane, while the proapoptotic protein Bax is present in the cytoplasm. Their expression maintains a balance, and these proteins antagonize each other, which is essential for maintaining normal cell morphology and function [29]. In response to certain factors, the expression of Bax is increased and translocates and inserts into the outer mitochondrial membrane to form large channels; moreover, the expression of Bcl2 is inhibited, and the stability of the mitochondrial membrane is damaged [30]. Cytochrome C and other proapoptotic factors are released to activate caspase3 and initiate the caspase cascade, leading to programmed cell death [29–31]. Cardiomyocytes have low tolerance for the inflammatory response, and an enhanced inflammatory response can initiate the caspase cascade, leading to excessive apoptosis in cardiomyocytes, which can lead to cardiac injury and an excessive decline in cardiac function. Vaspin reduces sepsis-mediated cardiac inflammation. To further explain the mechanism, we examined the effect of vaspin on myocardial apoptosis. The results showed that vaspin treatment significantly reversed the imbalance in Bax/Bcl2 expression and reduced the proportion of Caspase3 expression and TUNEL-positive cells. These results suggest that vaspin alleviates cardiac injury and cardiac dysfunction and may be related to protecting against cardiomyocyte apoptosis.

KLK7 is a member of the tissue kallikrein-releasing enzyme gene family and can be expressed in a variety of tissues and organs. Studies have shown that KLK7 is involved in the progression of multiple systemic diseases, including skin diseases, tumors, and prostatitis [32]. KLK7 is the first downstream signal of vaspin, and vaspin can significantly decrease KLK7 expression, thus inhibiting its downstream signal and ameliorating various diseases [6]. In addition, previous studies have found that vaspin was a valuable predictor of heart failure hospitalization during patients with acute myocardial infarction [33]. Our results also found that treatment with vaspin improved cardiac dysfunction, but whether this effect is mediated by KLK7 is still unclear. We further found that cardiac KLK7 levels were significantly inhibited in sham mice and CLP mice after vaspin pretreatment, suggesting that vaspin may be more likely involved in regulating septic-induced cardiac injury related to KLK7. Therefore, the regulatory effect of vaspin on sepsis progression was examined in KLK7-/- mice. The results showed that KLK7 knockout improves inflammation response, but still higher than the sham+KLK7-/- group. And vaspin-mediated regulation of survival, cardiac dysfunction, cardiac inflammation, and myocardial apoptosis in septic mice was masked by KLK7 knockout. These results suggest that vaspin-mediated regulation of sepsis-induced cardiac injury is mediated by KLK7.

In summary, our data showed for the first time that vaspin alleviates cardiac inflammation and protects against cardiomyocyte apoptosis by inhibiting KLK7 expression and alleviates sepsis-induced myocardial injury and cardiac dysfunction. Vaspin/KLK7 axis may be a potential therapeutic agent for treating septic cardiac injury, and KLK7 may play an even more important role.

## Data Availability

We confirm that all the data in our study could be freely available to scientists, except for commercial purposes.

## Supplementary Materials

Supplementary Materials Supplemental S1: effects of KLK7 deletion on sepsis-induced cardiac inflammation. mRNA expression levels of MCP-1, IL-6, IL-17, TNF-*α*, and IFN-*γ* were detected. *N* = 6 in each group. ^∗^*p* < 0.05 vs. the CLP + WT group. ^#^*p* < 0.05 vs. the Sham + KLK7 − /− group.
