# Supplementary material for: Vaspin Alleviates Sepsis-Induced Cardiac Injury and Cardiac Inflammation by Inhibiting Kallikrein 7 in Mice
Source: Mediators Inflamm. 2022 Jul 15;2022:1149582. doi: 10.1155/2022/1149582 (PMC9307398; doi:10.1155/2022/1149582)
Supplement: Supplementary Materials — Supplemental S1: effects of KLK7 deletion on sepsis-induced cardiac inflammation. mRNA expression levels of MCP-1, IL-6, IL-17, TNF-α, and IFN-γ were detected. N = 6 in each group. ∗p < 0.05 vs. the CLP + WT group. #p < 0.05 vs. the Sham + KLK7 − /− group. [file 1149582.f1.pdf]

Supplemental materials

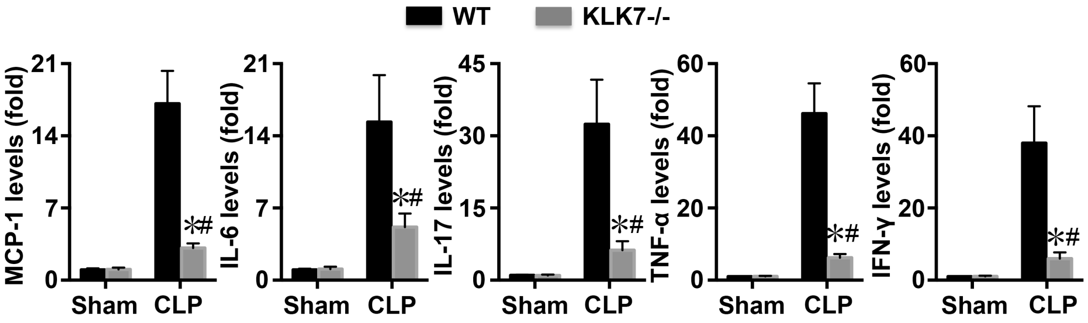

Supplemental S1: Effects of KLK7 deletion on sepsis-induced cardiac inflammation. mRNA expression levels of MCP-1, IL-6, IL-17, TNF-α, and IFN-γ were detected. N = 6 in each group. * $p < 0.05$ vs. the CLP + WT group. # $p < 0.05$ vs. the Sham + KLK7-/- group.
